# Supplementary material for: Are carbon emissions trading and green financial instruments synergistic? -Comprehensive quantitative research based on content analysis
Source: PLoS One. 2024 Mar 7;19(3):e0298601. doi: 10.1371/journal.pone.0298601 (PMC10919601; doi:10.1371/journal.pone.0298601)
Supplement: S1 Data — (ZIP) [file pone.0298601.s001.zip › Data/地级市Z_F工作报告.zip!/╕ú╜¿/╒─╓▌╩╨/└┤╫╘╣1⁄2╓┌║┼í╛┬φ┐╦╩2╛▌═°í┐_2007.docx]

各位代表：

现在，我代表市人民政府向大会作工作报告，请予审议，并请市政协委员和其他列席人员提出意见。

一、过去六年的政府工作

本届政府任期六年来，在省委、省政府和市委的正确领导下，在市人大法律监督、工作监督和市政协民主监督下，我们坚持以邓小平理论和“三个代表”重要思想为指导，全面落实科学发展观，加快构建社会主义和谐社会，积极推进海峡西岸经济区建设，经过全市人民的共同努力，“十五”计划顺利完成，“十一五”规划开局良好，改革开放和现代化建设取得重大成就。

六年来，经济实力明显增强。全市地区生产总值累计增长85.8%，年均增长10.9%；财政总收入累计增长184.81%，年均增长19.06%。工业强市成效显著，三次产业比例由30.6∶32.1∶37.3优化为23.1∶42.6∶34.3，规模工业产值增长2.7倍，“3+4”产业占规模工业比重83.2%。农林牧渔业总产值增长29.9%。建成一批专业批发市场，引进一批新型商贸零售和物流配送项目，服务业增加值年均增长10.4%，社会消费品零售总额年均增长10.3%。

六年来，基础设施日益完善。全社会固定资产投资累计完成1018亿元，年均增长12.1%。漳诏、漳龙高速公路建成通车，新建高等级公路231公里、二级公路235公里，建设农村水泥路3844公里，完成任务91%。招银港区六个万吨级泊位建成投入营运，漳州港成为全省第四个千万吨级港口、全国首批对台“三通”直航试点口岸。电力装机容量新增400万千瓦。厦大漳州校区、市医院门诊大楼等一批社会事业项目投入使用。完成城市整体规划修编，中心市区建成区面积由32平方公里扩大到43平方公里，城市化水平达到40.1%。荣获“中国优秀旅游城市”称号。

六年来，改革开放持续深化。以税费改革为重点的农村改革进展顺利，全面取消农业税、特产税、屠宰税和统筹提留，减轻农民负担11.07亿元。集体林权制度改革明晰产权任务全面完成。在全省率先完成农村信用社统一法人改革、国有控股上市公司的股权分置改革，国有企业完成新一轮改革改制。成立行政服务中心，建立经评审的最低价中标制度。对外开放进一步扩大，累计实际利用外资（历史口径）42.34亿美元，其中实际利用台资22.3亿美元。外贸出口103.6亿美元，年均增长31.6%。漳台农业合作不断拓展，海峡两岸花博会由区域性展会升格为全国性的花卉盛会，台湾农民创业园获国台办、农业部批准设立，东山对台小额贸易连续多年位居全省前列。侨务、对台、外事工作取得新成绩，迎接国民党荣誉主席连战回乡谒祖活动获得圆满成功。

六年来，群众生活不断改善。完成106件为民办实事项目，不断改善人民群众生产生活条件。城乡电网升级改造全面完成。农村中小学危房改造项目完成336个，新建和修缮面积达46.95万平方米。建成经济适用房20.8万平方米，城市居民人均住房面积27平方米，增加42%。强化就业和社会保障工作，累计新增城镇就业21.5万人，转移农村富余劳动力37.1万人，城镇登记失业率控制在4%以内。企业离退休人员养老金和下岗失业人员失业金100%按时足额发放，农村养老保险覆盖面不断扩大，城乡低保户实现应保尽保，农村住房保险全面推行。社会救助体系进一步完善，受灾群众、农村五保供养户、城乡因病致贫家庭和困难大学生得到及时救助。民族、宗教、老区、妇女儿童、老龄、残疾人等工作取得新成效。

六年来，社会事业全面进步。大力实施科教兴市战略，高新技术产业产值增长6倍，荣获全国科技进步先进城市。教育“两基”巩固提高，高中阶段毛入学率提高37.3%，高等教育、职业教育、民办教育发展加快。文化体育事业取得新成绩，市区香港路--台湾路历史街区荣获联合国亚太地区文化遗产保护奖，5个县（市、区）跨入全国体育先进县行列。疾病预防控制体系和公共卫生医疗救治体系基本建成，镇级卫生院建设步伐加快，全市新型农村合作医疗覆盖面达60%。人口与计生工作取得新成效，长泰、龙海、芗城荣获全国计生优质服务县（市、区）。耕地总量连续八年实现占补平衡，环境污染和生态破坏得到有效控制。严厉打击各类违法犯罪活动，“四五”普法教育顺利完成，社会治安综合治理成效明显。国防动员和双拥优抚工作持续加强，荣获全国双拥模范城市“三连冠”称号。群众性精神文明创建活动广泛开展，荣获全国创建精神文明工作先进城市。人事、编制、气象、防震、档案、方志等工作都取得新成绩。

六年来，政府自身建设得到加强。认真贯彻落实市委各项决策，认真执行市人大及其常委会的决议，多途径征求人大代表和政协委员的意见建议，共办理人大代表议案158件、建议878件和政协提案1202件，办理结果满意率进一步提高。认真实施行政许可法和全面推进依法行政实施纲要，开展相对集中行政处罚权工作。完善重大决策咨询、听证、人民建议征集制度，建立社情民意反映制度。开通“中国漳州”政府门户网站、“政风行风热线”，电子政务不断完善，政务公开全面推进。深入开展效能建设，推行政府绩效评估。建立突发公共事件应急处理机制。强化行政监察、审计监督，廉政建设和反腐败工作取得新成效。

2006年，是我市发展进程中不平凡的一年。面对接踵而至的强台风、强暴雨等自然灾害，我们坚持一手抓抗灾救灾，一手抓经济发展，夺取了抗灾救灾和经济建设的双重胜利，全面完成了市十三届人大七次会议确定的各项目标任务。实现地区生产总值714.93亿元，比上年增长12.5%；财政总收入63.75亿元，增长26%，比2003年翻一番，其中地方级财政收入35.06亿元，增长30%；社会消费品零售总额258.35亿元，增长12.4%；城镇居民人均可支配收入12511元，增长11.3%，农民人均纯收入5071元，增长8.1%；金融机构本外币各项存款余额531.03亿元，同比增长20.3%，本外币各项贷款余额345.88亿元，同比增长17.67%。呈现七个可喜的亮点：

工业发展跃上新台阶。实现工业总产值982.7亿元，增长22.7%，其中规模工业产值786亿元，增长27.6%。建设投资500万元以上的工业项目776个，其中投资5000万元以上的项目150个，上亿元的项目60个；已投产的项目351个，其中投资5000万元以上的项目41个，上亿元的项目19个。新增规模工业企业278家，灿坤家电、凯西不锈钢、联盛纸业、大众兴纺织等一批重点项目建成投产。年产值超亿元企业133家，增加37家。新增5件中国驰名商标、15件著名商标、17项福建名牌产品、7项国家免检产品。

新农村建设取得新进展。编制实施新农村建设规划纲要，农村经济稳步发展，实现农林牧渔业总产值294.39亿元，增长3.9%，粮食生产超额完成省政府下达的任务。新增无公害农产品24个、绿色食品19个。转移农村富余劳动力近8万人，启动“千村整治、百村示范”工程，30家重点支持的乡镇卫生院改造提升工程全部完成，“六千”水利工程投资完成年度计划的141.3%，农村水泥路建成1006公里，全面完成电力、移动电话、广播电视“村村通”工程。

固定资产投资再创新高。全社会固定资产投资完成239.44亿元，增长30.5%，增幅为1999年以来最高。城镇以上工业投资增长41.5%，工业投资占全部城镇投资比重比全省高出近20个百分点。全市181个在建重点项目完成投资134.6亿元，占全社会固定资产投资的56.2%，比上年提高5.3个百分点。龙厦铁路漳州段等一批重点项目开工建设。

对外经贸合作实现新突破。新批注册合同外资8.77亿美元，增长25.88%；验资到资4亿美元，增长29.2%；引进投资千万美元以上大企业58家。日产游艇正式落户，艾默生、沃尔玛投产运营，实现引进世界500强零的突破。外贸出口29.8亿美元，增长15%。漳台经贸合作取得新成效，新批台资企业136家，验资到资2.3亿美元，增长38.9%，台湾百强企业台湾玻璃正式落户漳浦。

城市建设展现新面貌。荣膺国家园林城市称号，“创卫”工作有序开展，“创模”通过国家技术核查。市区江滨路等14个项目竣工，液化管道天然气、西洋坪大桥等项目有效推进。完成新华南北路等市区道路综合整治改造，实施海峡西岸城市群夜景工程，市区内林引水、浦头港排涝工程投入使用，三湘江整治加快推进。在全省率先编制完成全市风景名胜区体系规划。房地产业较快发展。

社会各项事业得到新发展。荣获首届省级文明城市称号。新增省级以上高新技术企业21家，全市高新技术企业产值220亿元，增长35%。落实农村义务教育“两免一补”政策，受惠学生50万人，高中阶段教育毛入学率69.3%，比上年提高近10个百分点。不断繁荣艺术创作，活跃对外文化交流。新增芗城、漳浦、南靖三个新型农村合作医疗试点，参合农民达105.28万人。第十三届省运会我市奖牌数和团体总分均居第四名，成功举办市第五届农运会、第七届老运会。人口与计生工作一类先进县比例居全省第二位，国优、省优县比例居全省第三位，被确定为全国人口与计生工作综合改革及新机制建设试点市。九龙江漳州段水质达标率95.5%，城市环境质量考核位居全省前列。“平安漳州”创建活动深入开展，社会安定稳定，公众安全感满意率达95%，公安机关多项工作继续保持在全省全国的领先行列。

抗灾救灾和家园重建扎实有效。去年我市连续遭受三次强台风和持续强降雨的袭击，损失之大前所未有。各级党委、政府靠前指挥、果断决策，全市上下全力以赴、奋力抗灾，驻漳部队冲锋在前、英勇奋战，社会各界积极援助、扶危济困，把人员伤亡和经济损失降到最低程度，受灾群众得到妥善安置，江河水库安全渡汛，水毁工程迅速抢修，灾后生产及时恢复，家园重建扎实推进，投入救灾资金2.99亿元，23304户重建户全部搬入新居。

各位代表！六年的发展，六年的进步，是省委、省政府和市委坚强领导的结果，是全市人民团结拼搏的结果，也是历届政府打下良好基础的结果。在此，我代表市政府向辛勤工作在各行各业的广大干部群众，向给予政府工作大力支持的各级人大代表、政协委员、各民主党派、工商联、离退休老同志、各界人士，向为漳州改革发展稳定做出积极贡献的中央、省驻漳单位和驻漳部队、武警官兵，向所有来漳的投资者和建设者，向关心支持漳州发展的港澳台同胞、海外侨胞和国际友人表示诚挚的感谢！

回顾六年的发展历程，我们深刻体会到，做好政府工作，加快漳州发展，必须始终坚持市委正确领导，凝聚发展合力；始终坚持突出第一要务，强化发展意识；始终坚持主动融入大局，找准发展定位；始终坚持不断解放思想，创新发展理念；始终坚持推进改革开放，激发发展活力；始终坚持为民惠民利民，共享发展成果。这些经验弥足珍贵，要在今后工作中认真坚持。

六年的发展成绩来之不易，但我们也要清醒看到，在前进中还存在许多困难和问题。主要表现在：经济总量、综合实力、发展速度与我市拥有的区位和资源条件不相称，经济整体素质和竞争力与先进地区相比仍有明显差距；支撑经济快速发展的大型骨干项目仍然偏少，投资扩张能力不足；区域发展不平衡，县域经济比较薄弱，县乡财政仍较困难；自主创新能力亟待提升，具有影响力的名牌品牌不多；农村防灾减灾体系仍较脆弱，农村生产生活环境有待进一步改善，农民持续增收难度加大；城乡就业结构压力尚未得到根本缓解，社会弱势群体生活仍较困难，一些涉及群众切身利益的问题有待进一步解决；政府自身建设还存在着一些薄弱环节，社会管理和公共服务需要进一步加强等等。在今后的工作中，我们将高度重视，认真加以解决。

二、今后五年的奋斗目标和主要任务

今后五年是我市加快发展、实现赶超的关键时期。我们已站在一个新的历史起点上，自身优势在展现，发展活力在迸发，创业机会在增多，干部群众干事创业的热情高涨，特别是国内外宏观环境变化，总体上仍然有利于实现又好又快发展。我们要珍惜和用好战略机遇期，立足新起点、瞄准新目标、拓展新思路、实现新作为，纵向争翻番、横向争位次、对内争效率、对外争形象，努力实现经济社会跨越式发展。

今后五年政府工作的指导思想是：坚持以邓小平理论和“三个代表”重要思想为指导，全面落实科学发展观，加快构建社会主义和谐社会，认真贯彻党的十六大以来各项方针政策和省、市党代会部署，围绕“融入海西、依港立市，农业固基、工业强市，五缘六求、开放活市，统筹协调、科教兴市”的总体思路，把省委“重在持续、重在提升、重在运作、重在实效”的实践要领贯穿于各项工作中，全力推进经济社会又好又快发展，为打造海峡西岸港口大市、工业强市、生态名市、和谐漳州奠定扎实基础，更好发挥漳州在海西建设中的骨干作用。

根据以上指导思想和总体要求，今后五年全市发展的主要奋斗目标是：全面建设小康社会，贯彻落实《福建省建设海峡西岸经济区纲要》，主要经济指标增幅高于全省平均水平，经济综合实力更强，地区生产总值突破1200亿元，财政总收入突破160亿元，缩小与省内先进地区的发展差距；人民生活水平更高，发展更协调，生态更良好，社会更和谐；全面完成“十一五”规划目标，把我市建设成为与厦门特区互动、对台合作紧密、对外开放活跃的生态工贸港口城市，建成海峡西岸经济区的现代制造业基地、创新型城市和重要增长极，建成辐射全国的优质食品供应基地、两岸经贸合作基地和农产品加工出口基地，建成东南沿海重要的出海通道、连接珠三角、闽西南和赣南腹地的重要交通枢纽，建成资源节约型和环境友好型社会的示范区。

实现以上目标，我们一定要坚持解放思想，实事求是，与时俱进；一定要增强不进则退、慢进也是退的危机感和使命感；一定要激发加快发展的激情与责任，开拓进取，勇于拼搏；一定要强化竞争意识、市场意识和服务意识；一定要落实创新、创造、创业举措，力争在海峡西岸经济区建设中谋求更大作为。要着力在六个方面建设取得重大进展：

建设港口大市取得重大进展。按照建成国家重要综合运输枢纽港的目标，加快推进“一港七区”开发建设，积极参与厦门港发展分工，谋求招银、后石、石码港区新发展；统一规划环东山湾各港区，加快古雷、云霄、东山和诏安港区开发建设步伐。建成龙厦铁路漳州段、厦深铁路漳州段、厦漳跨海大桥，建设招银、古雷、东山港区铁路支线，形成各县（市、区）连接港口的快捷运输网络。加快建设九龙江口、东山湾两个临港产业聚集区，大力发展临港大型加工制造业。扶持发展港口物流业，积极拓展滨海旅游业、远洋渔业和海上运输业。至2011年，力争临港工业产值比2005年翻两番以上，港口吞吐量6000万吨以上，集装箱80万标箱，海洋产业增加值达220亿元。

建设工业强市取得重大进展。大力实施项目带动战略，优化产业结构，转变增长方式，增强自主创新能力和市场竞争能力。努力打造“3+4”产业基地，加快发展配套型企业，延伸产业链，壮大产业集群。加大对世界500强、台湾百大企业的招商力度，争取实现新突破。鼓励帮助一批优势企业上市融资，培育壮大一批企业“小巨人”。大力实施品牌战略，增强工业经济竞争力。至2011年，累计实际利用外资25亿美元（验资口径）、固定资产投资超过2000亿元，全市规模工业产值突破2000亿元，形成一批产值超50亿元、100亿元、纳税超亿元和拥有中国驰名商标、中国名牌产品的企业群体。

建设中心城市取得重大进展。加强中心城市和九龙江两岸城镇群规划管理，加快桥南片区、长洲片区、角美片区、金峰片区建设，推动中心城市“东移、西进、南扩、北延”。强化中心城区交通枢纽、商品集散和信息流通功能，积极发展服务业，形成旺盛的城区人流、物流、资金流，壮大城区经济。加强中心城区与县域城关在基础设施和商贸等方面的对接，带动小城镇建设，形成协调发展、层次分明的城镇体系。至2011年，力争中心城区建成面积超50平方公里、人口超过50万人，全市城市化水平达50%以上。

建设新农村取得重大进展。按照“生产发展、生活宽裕、乡风文明、村容整洁、管理民主”的总体要求，坚持工业反哺农业、城市支持农村和多予少取放活的方针，加大对“三农”投入，组织实施新农村建设五年规划。大力发展现代农业，加强农村基础设施建设，深入开展家园清洁行动，加大农村富余劳动力转移力度，加快培育新型农民，加强基层组织建设，推进农业持续增效、农民持续增收、农村更加和谐。至2011年，全市农业增加值达到185亿元以上，农民人均纯收入超过7000元，累计转移农村富余劳动力35万人以上，有线电视入户率38%以上，安全饮用水合格率达100%，卫生厕所普及率50%以上。

建设生态名市取得重大进展。巩固提升国家园林城市和中国优秀旅游城市创建成果，积极创建国家环保模范城市、卫生城市、生态示范城市。加强保护江河源头区、水源涵养区、饮用水源保护区和渔业水域，治理水土流失，加大生态公益林、湿地和沿海防护林体系建设。有序开发和保护旅游资源，突出滨海火山、开漳文化、宋城遗迹、土楼民俗四大品牌，打造三大特色旅游带。至2011年，全市森林覆盖率稳定在62%以上，城市建成区绿化覆盖率达45%以上，主要流域水环境质量达标率95%以上，中心城市空气质量优于国家二级标准，万元GDP能耗下降12%以上。

建设和谐漳州取得重大进展。按照 “五个统筹”要求，改善科教、文化、卫生、体育等社会事业基础设施。改革文化体制，培育文化品牌。稳定低生育水平，提高人口素质。努力实现城乡居民收入水平与经济发展速度相协调。着力解决人民群众最关心、最直接、最现实的问题。至2011年，全市高新技术产业增加值占规模以上工业增加值的比重提高到35％以上，高中阶段毛入学率达80%以上，建立和完善新型社会救助体系，城乡居民最低生活保障实现全员覆盖，基本医疗保险覆盖到所有城镇居民，新增就业25万人，城镇登记失业率控制在4%以内，城镇居民人均可支配收入突破2万元。

三、雷厉风行、奋发有为做好2007年工作

2007年是党的十七大召开的喜庆之年，也是全面落实市第九次党代会战略部署的开局之年，做好今年工作意义深远、责任重大。主要预期目标为：全市生产总值增长13 %；规模工业增长26%；财政总收入完成80亿元，增长25.5%；固定资产投资增长25%；社会消费品零售总额增长13%以上；外贸出口增长10%；实际利用外资力争增长12.5%；城镇居民人均可支配收入增长10.8%，农民人均纯收入增长6.5 %；城镇登记失业率控制在4%以内；人口自然增长率控制在6.66‰以下。单位生产总值能耗降低2.6%。在执行中，力争更好更快。

完成以上预期目标，要突出抓好以下八项重点工作：

（一）充分发挥港口优势，扎实推进依港立市

推进港口大开发。主动融入厦门湾，抓紧全市港口总体规划和相关专项规划的修编和实施。多方筹措资金，加快配套项目建设。努力创造条件促进石码、冬古、宫口三个二类口岸并入一类口岸，争取早日对外开放。加快招银港区7#泊位、古雷一德石化码头投入生产运营，推进招银港区8#、9#泊位建设，开工建设东山石化码头，做好龙池码头一期工程、古雷港区陆域扩建工程、古雷10万吨级航道和厦门港招银航道一期扩建工程等项目前期工作。

培育临港大工业。着力引进一批产业主导性强、带动力大的冶金、石化、能源等临港工业大项目，推动临港产业集聚。加快台湾玻璃、凯景新型精品涂镀层板投建投产，促成福欣特钢、后石电厂三期7号机组尽早获得核准并开工投建。继续开展后石电厂8、9、10三台机组、古雷火力风力发电、六鳌风力发电三期、东山乌礁湾风力发电三期及核电等重大临港工业项目前期工作。

构筑疏港大通道。加快建设龙厦铁路漳州段，开工建设厦深铁路漳州段、厦漳跨海大桥。做好厦成高速公路漳州段、沈海高速公路（一纵）漳州段拓宽工程、沈海高速公路复线（二纵）漳州段、市区高速公路连接线、招银港区至沈海高速连接线、古雷港区至武平高速公路漳州段、漳州至永安高速公路漳州段的前期工作，力争早日动工建设。争取东山港区高速公路连接线列入国家和省项目计划。实施国省干线公路改造工程，加快省道东东线等25个交通重点项目建设，推进连接九龙江河口南北岸通道和南部、西部片区的山海通道建设，形成与港口匹配、快速便捷的集疏运网络。

（二）加快壮大产业规模，扎实推进工业强市

以推进项目带动为龙头。努力提高工业项目在重点项目中的比重，提高重点项目在固定资产投资中的比重。今年安排市重点项目322个，其中在建重点项目231个，年度计划投资161.2亿元；预备项目91个，安排重大前期项目74个。要按照“前期项目促报批，在建项目促进度，竣工项目促投产”的要求，进一步落实责任制，确保项目建设进展顺利。天龙达电子等101个新开工项目要抓紧动工建设，厨师食品等49个计划竣工项目年内要确保建成投产，市区电网建设等81个续建项目要加快推进。要高度重视土地报批和工业项目前期工作，催生源源不断的后续项目，形成规划一批、储备一批、引进一批、开工一批、投产一批的良性投资机制。

以壮大民营经济为突破口。抓紧编制《漳州市中小企业发展规划》，落实扶持民营经济发展政策，营造公平、公正、竞争、有序的发展环境，帮助民营企业解决融资、用地、引进人才、用工等方面的问题，在全社会形成“想创业、敢创业、会创业、创大业”的发展氛围。鼓励和引导民营企业实行股份制改造，完善激励机制、竞争机制和内部监督机制，着力提高企业素质，推动民营经济发展上新台阶。

以促进产业升级为目标。以信息化带动工业化, 推动传统产业升级换代，加快发展高新技术产业和新兴支柱产业。壮大“3+4”产业，做大做强13个产业集群。扶持万利达、立达信、漳州（金龙）汽车、福环汽车、钟表工业和龙翔草酸等专业生产基地发展壮大，发挥龙头企业的集聚带动作用，促进专业化分工与协作，培育和引进一批“专、精、特、新”的配套企业，完善上下游产业，扩大产业规模，形成产业整体竞争优势。

以提高企业竞争力为重点。全力实施“千百工程”，力争投建500万元以上工业项目1000个，总投资额上亿元的项目100个。引导企业以市场、资本、品牌为纽带，进行资产重组，形成一批具有核心竞争力的企业集团和一批具有专业竞争力的企业群。加快国有企业、二轻集体企业改革，逐步建立健全现代企业制度。提升上市公司质量，鼓励帮助有条件的企业上市融资。突出抓好299个新增产值1000万元以上工业经济支撑项目，壮大年销售超亿元的企业群体，扶持税收超500万元的成长型企业，加速优质资源向优势企业集聚，打造一批“旗舰型”企业。

以实施品牌战略为导向。坚持“政府引导，企业主导，中介辅导”，建立健全品牌培育工作机制，落实各项扶持奖励措施，对全市重点产品进行建档跟踪，加强分类指导。鼓励企业扎实开展品牌兴企、质量兴企活动，按照国际标准加强质量管理，努力创名牌、办名厂、出名品，借助品牌效应加快扩张。各县（市、区）要重点选择一批科技含量高、市场潜力大、发展前景好的产品予以扶持培育，鼓励企业争创一批中国驰名商标和中国名牌产品。

以工业园区建设为载体。工业园区建设是推进工业强市的重要载体。要继续整合省级以上开发区资源，优化工业发展布局，提高开发区产业承载能力。漳州开发区、龙池开发区要发挥好基础设施完备、品牌效应突出的作用，在经济发展中起带头示范效应，并努力成为漳州新的增长极。古雷开发区要加快以基础设施配套为主的开发建设，争取引进一批临港产业大项目。积极筹建IT产业园区，推动产业结构调整升级。漳浦集控区要抓紧开工建设，诏安集控区要抓紧前期的申报准备工作。其他工业园也要加快建设步伐，发挥应有作用。

（三）积极拓展招商引资，扎实推进开放活市

提高外引内联水平。加强外资投向引导，依托现有产业链和产业集群招商，结合产业结构调整升级，着重引进先进适用技术和现代管理经验。积极筹办首届“钟表论坛”，充分利用“4·9”、“6·18”、“9·8”、“11·18”等招商平台，加大项目对接和引进力度，力争重大项目引进取得新突破。对重大招商活动签约项目要加强跟踪服务，抓紧报批升级，对投资千万美元以上的项目要加快筹建步伐。积极组织到省内外举办招商推介会，争取引进一批内联项目。枋洋水利枢纽工程力争尽早开工。切实加强后招商工作，坚持服务至上，营造良好投资环境。着力转变外贸出口增长方式，培育出口品牌，积极应对国际贸易壁垒，推动外贸出口持续增长。

扩大漳台经贸合作。发挥五缘优势，拓展六求作为，彰显台胞祖籍地作用，着力在产业对接、农业合作、民俗文化和旅游等方面加强交流。支持现有台资企业发展，保护台商合法权益。积极承接台湾产业转移，力争在引进大项目上有新突破。发挥海峡两岸农业合作试验区的先行优势和台湾农民创业园的窗口示范和辐射作用，争取设立对台农产品物流加工保税区，不断提升漳台农业合作水平。继续办好海峡两岸花卉博览会暨农业合作洽谈会，精心筹办海峡两岸（福建东山）关帝文化旅游节和“开漳圣王”（中国·云霄）文化节，拓宽漳台经贸文化交流渠道，推动漳台民间往来。同时要加强与港澳的经贸合作，做好海外侨胞、归侨侨眷的工作，凝聚侨心、汇集侨智、发挥侨力。

（四）大力增强自主创新，扎实推进科教兴市

加快科技创新进程。推进创新型城市建设，实施科技产业、创新服务体系建设、科技引导示范三大工程，建设一批为重点行业、重点企业提供创新服务的公共平台，重点扶持智能小家电、视听产品、光电照明、钟表等行业建立技术中心或研发中心。深化与国内外知名高等院校合作，增强“6·18”项目成果对接能力，抓好省市共建的高新技术创业孵化基地建设，构筑技术创新合作平台，努力培植一批自主创新型示范企业。加强自主知识产权保护，重奖有功科技人才。加快高新技术产业园区和民营科技园区的建设步伐，争取新发展高新技术企业12家以上，高新技术产业产值250亿元以上。

优先发展教育事业。坚持教育优先发展战略。促进义务教育均衡发展，全面落实农村义务教育“两免一补”政策。继续调整城乡中小学布局，加快农村寄宿制学校建设，建立农村中小学危房改造长效机制，推进“双高普九”工作；大力发展高中阶段教育，确保高中阶段毛入学率达71%以上。发挥厦大漳州校区、漳州师院的带动作用，推进漳州高教园区建设，支持漳州职业技术学院争创国家级示范校。创新职业教育办学模式，支持办好漳州卫生职业学院、漳州城市职业学院。鼓励社会力量办学，引导民办学校健康发展。

加大人才引进力度。加强人力资源建设，调整优化人才结构。抓好人才培养、引进和使用三个环节，认真开展干部培训工作，抓紧修订人才引进政策，完善人才引进机制，营造“重才、爱才、惜才、育才”的良好社会环境。坚持产业聚才、项目引才，发挥企业引才、用才的主体作用。加快建设一批博士后工作站，引进一批掌握核心技术的人才、复合型科技人才和高技能人才。鼓励实行技术入股、管理入股，充分调动人才创业积极性。

（五）更加重视“三农”工作，扎实推进新农村建设

加快发展现代农业。切实增加财政对“三农”的投入。稳定粮食播种面积，提高粮食综合生产能力，推进农业固基。以水仙花、榕树盆景、绿化苗木为主要品种的花卉产业，要借助荷兰、台湾地区的技术、市场，不断提高自己的栽培技术和营销策略，拓宽销售渠道，提高国内外市场份额和经济价值。以香蕉、蜜柚、柑桔、枇杷、青梅为主的水果产品，要引进台湾的先进栽培、包装、保鲜技术，改良品种，科学管理，提高品种质量，创立漳州名果品牌，打开国内外市场销路。以捕捞、养殖、加工为一体的水产品，要针对养殖条件、市场变化，摸索捕捞、养殖新技术，深化加工，提高水产品的附加值，增加渔民收入。要围绕做大做强花卉、水果、水产、蔬菜、茶叶、畜牧、食用菌等特色产业规模这篇文章，培育一批特色明显、类型多样的专业村、专业镇。加强农产品质量安全体系建设，大力发展无公害农产品、绿色食品、有机食品，打造一批农产品品牌。扎实推进农业产业化经营，培育一批市场竞争力强、辐射带动面广的龙头企业群体和新型农民专业合作组织。鼓励支持现代农业技术成果的应用推广，实施一批农业“五新”示范项目。抓好农村劳动力培训和劳务派遣工作，促进农民向城镇和二三产业转移，增加农民非农收入。

加强农村基础设施建设。积极开展农田水利建设，抓好农业综合开发和“六千”水利工程，加快建设标准化农田。继续实施农村“年千公里”水泥路建设工程，基本实现村村通水泥路。实施农村户用沼气工程。加强防汛和排涝工程体系、气象预警报系统和防汛指挥系统建设，加强动植物病虫害防控体系建设，完善森林火灾和海上渔业安全预警监测、救助指挥系统建设。重视新村规划建设，推进“千村整治、百村示范”和“三清六改”工程，积极开展“家园清洁行动”和“创绿色家园、建富裕新村”活动。

深化农村综合改革。积极稳妥推进乡镇机构改革，完善农村义务教育管理体制，深化粮食流通体制和集体林权制度改革，推进小型水利设施、海域和内陆水域有偿使用制度的实施。建立完善农村金融服务体系，加大对“三农”信贷支持力度。创新农村工作机制，着力解决村级组织无活动场所问题，充分发挥农村三级核心网络作用，落实农村“一事一议”，健全村务公开和民主管理制度，加强农村“六大员”培训和管理。确保全国农业普查工作顺利开展。

（六）突出建设管理并重，扎实提升城市形象

拓展城市发展新空间。继续实施“建设新区、开发沿江、改善旧城”的城市发展战略，抓好年度投资10亿元的26个城建项目建设，重点实施市区“十一路二江一桥一堤一气”工程，不断撑大城市框架。加强对房地产市场的监管和调控，引导房地产业健康发展，抓好经济适用房、廉租房和安置房建设，力争惠民花园首期开盘预售。大力发展城市经济，筹建会展中心，积极发展现代物流业，推广新型营销方式，推进明发商业广场和4家五星级酒店建设，繁荣服务业。开发滨海生态旅游资源，加大旅游宣传促销力度，打响中国优秀旅游城市品牌，增强城市吸引力。

打造城市发展新亮点。加强西溪南北岸城市景观规划，提前做好土地控规，为营造“一江两岸”城市景观打好基础。继续实施夜景工程。加大国家卫生城市和国家环保模范城市创建力度，积极创建国家生态示范城市。深化“爱我漳州”活动，努力建设滨海特色突出、山水园林相间、生态环境优美的魅力城市，促进城市环境和人文环境的和谐统一，创造良好的人居环境。

推行城市管理新机制。加强城市管理综合执法，加大对“脏、乱、差”和违规建筑的治理力度。积极运用市场机制，多渠道筹措城市建设资金。运作好土地收储中心和城投公司，规范土地一级开发，加强土地出让成本核算，严格推行经营性土地招拍挂，执行工业用地出让最低价标准，提高土地利用效益，增强城市建设投融资能力，促进城市建设良性发展。继续推进市政、绿化、环卫、公交等公用事业改革，加快九龙岭垃圾处理场产业化运作，对广告牌位、行业特许经营权进行公开竞标拍卖，增加无形资产收益。

（七）广泛激发创造活力，扎实推进县域经济发展

运用政策推动效应。坚持分类指导、整体推进、挖掘潜力、创新机制，激发县域经济发展活力。落实支持县域经济发展的各项政策，切实解决县域经济发展过程中出现的困难和问题。完善财政收入激励机制，调动各级培养财源积极性，积极争取上级支持，多方筹措资金减轻各级债务负担，努力增加各县（市）可用资金。加强县城和中心城镇的规划建设与管理，提高城镇化水平。积极创造条件，让基层和企业有更大的发展自主权。

发挥产业拉动效应。各县（市）要从实际出发，依托自身的比较优势，大力发展特色产业，扩大县域经济规模。培育各具特色的县域工业团组，因地制宜地发展劳动密集型产业，提升中小企业发展水平，形成“一县一业、一镇一品”的经济发展格局。发挥特色支柱产业的带动作用，增强地方吸纳技术、项目、资金、人才的能力。

策划造势驱动效应。以东南花都展厅为载体，精心筹办各类区域性、全国性商品交易会、展销会，力争一季一展会，促进经济活跃和商贸繁荣。支持县（市）依托产业基础和区位优势，办好各种推介会、展会、节会，营造浓厚的发展氛围。开展“工业发展年”、“项目建设年”、“平安企业年”等活动，形成竞相发展的良好态势。

强化市场带动效应。改造建设市区花卉交易中心、现代农产品综合批发市场、粮油饲料批发市场、汽车汽配交易城、药品市场、建材市场、家居市场等专业市场，规划建设农资仓储物流中心，抓好特色商业街、商业重点镇建设和社区商业“双进”工程试点工作，推进“万村千乡”和“双百”工程建设，引导连锁店和超市加快向农村延伸发展，实现村村有“农家店”，形成物流旺盛的漳州市场。

（八）始终坚持以人为本，扎实推进和谐社会建设

更加重视民生改善。认真抓好总投资额约14.8亿元的31项为民办实事项目的落实，改善群众生产生活条件。高度重视零就业家庭的就业，深入开展再就业援助行动，拓宽就业渠道，促进就业和再就业。扶持少数民族地区加快发展。继续完善对口扶持、干部下派驻村和市领导挂钩贫困村、示范村制度，采取整村推进、造福工程、产业扶贫、小额贷款等扶贫措施，帮助贫困地区和低收入人口脱贫致富，抓好大中型水库农村移民后扶政策的落实。积极探索建立失地农民养老、医疗保障有效途径。加强灾害救助、社会救济、社会救助三大体系建设，认真解决革命“五老人员”、农村“五保户”、计生双女户、特困户、库区移民、城乡低保户、下岗失业人员和残疾人的实际困难。重视社会福利和慈善事业发展，抓好福康医院综合病房大楼和市老年护理中心的建设。

更加重视协调发展。全面加强人口与计生工作，强化计生队伍建设，发挥各级计生协会的作用，实施关爱女孩行动，落实计生帮扶措施。加快建立新机制，重点整治流动人口管理不力、出生人口性别比偏高、常规措施拖欠、出生漏报漏统等问题，稳定低生育水平，提高出生人口素质。做好重大疾病的防控，积极改善城乡医疗卫生条件，推进城市社区卫生体系建设，加快乡镇卫生院的改造提升；全面推行新型农村合作医疗，努力解决农村“看病难”问题。不断繁荣文艺创作，强化文化市场管理，建设基层宣传文化中心户，加强文化遗产保护，做好土楼申遗工作。加快城乡体育设施建设步伐，广泛开展全民健身活动，不断提高新周期竞技体育水平。实施有线数字电视整体转换和20户以上广电村村通工程。深化群众性精神文明创建活动，开展以“八荣八耻”为主要内容的社会主义荣辱观教育，营造全社会讲诚信、讲友爱的风气。加强国防教育、人民防空建设、双拥共建、民兵预备役和优抚安置工作，争创全国双拥模范城“四连冠”。

更加重视平安建设。大力实施“五五”普法，进一步加强社会治安综合治理，依法严厉打击各种刑事和经济犯罪，刑事发案率进一步降低，公众对社会治安满意率达到95%以上，全市11个县（市、区）全部达到“平安县”标准。加强应急管理，完善处置预案。坚决纠正各种侵害群众利益的行为，进一步强化信访工作，引导群众以理性合法的形式表达利益诉求，努力化解社会矛盾。高度重视安全生产，强化“两个主体”意识，进一步落实安全生产责任制，深化道路、消防、渔业等重点行业的整治，加大执法检查力度，有效遏制重特大事故发生。整顿规范市场经济秩序，重点打击以制售假烟为主的制假售假违法行为。加强食品药品监督管理，确保人民群众饮食和用药安全。

更加重视生态和谐。严格执行国家土地政策，认真修编土地利用总体规划，优化基本农田和建设用地布局，完善建设用地定额标准，盘活存量，用好增量；建设标准厂房、多层厂房，实现节约和集约用地；加强土地整理，保持耕地总量动态平衡。着手编制水资源综合开发利用规划，合理配置水资源，实现水资源可持续利用。加强山地开发管理，综合治理水土流失和地质灾害隐患。切实转变经济增长方式，大力发展循环经济和节能环保产业，扶持发展资源回收再生产业，规划建设再生产业园区。落实市长环保目标责任制，强化城镇污水处理厂、垃圾无害化处理场等环境基础设施建设，严格执行环保“三同时”制度，综合治理工业“三废”，切实控制和削减排污总量。加快市区西区、龙海、东山污水处理厂和角美开发区污水处理厂二期工程建设，加强海洋环境和水资源综合整治，确保九龙江西溪水质达标率90%以上，北溪水质达标率达100%。

四、进一步提高政府的执行力和公信力

各级政府要组织广大干部认真学习《江泽民文选》，按照胡锦涛总书记强调的学习“四种知识”、提高“四个能力”和倡导“八个方面的良好风气”的要求，切实加强政府自身建设，创新工作机制，努力建设推进发展、体现人民意志的法治型、服务型、责任型、效能型政府和节约型政府。

勇担责任，为民利民。牢记“两个务必”，当好人民公仆，促使每一位干部肩上有担子、心中有责任，义不容辞地承担起发展一方经济、富裕一方群众、维护一方平安的责任。要敢于直面复杂的矛盾和困难的局势，勇于知难而进、迎难而上，锤炼锲而不舍、坚韧不拔的意志。要密切政府与群众的联系，以真挚的感情倾听群众的所思、所盼、所求，切实把心思和精力用在关注民生上，时刻把人民群众的安危冷暖放在心头。要努力提高社会管理水平，加强对涉及人民生命财产安全领域的监管，把有限的资金用在最需要发展的事业上，把新增财力主要用于解决就业、就学、就医、社会保障、社会治安、安全生产、环境保护等人民群众最关心、最直接、最现实的利益问题，让人民群众共享改革开放的成果。

雷厉风行，落实务实。大力倡导雷厉风行、奋发有为的工作作风，狠抓决策部署的落实。做到领导在一线指挥，问题在一线解决，业绩在一线创造。深化机关效能建设，完善政府绩效评估，推行问责制度，加大督查力度，切实提高政府执行力。政府各个部门都要围绕发展大局和工作中心，充分发挥职能作用，强化服务意识，关心支持基层工作，用心尽力地为基层、企业和群众服务。要按照“重在提请、主动服务”的要求，秉持亲民亲商理念，建立重大投资项目跟踪服务制度，为重大项目的投资者设立绿色通道，把投资者的事、企业的事当作自己的事来办，真心实意、想方设法支持项目的建设和企业的发展。深化行政审批制度改革，完善行政服务中心功能，加强电子政务建设，简化办事环节，压缩服务时限，促进行政服务提速增效。

依法行政，清廉清正。把依法行政贯穿于政府工作全过程，依法管理经济社会事务。健全科学民主的决策机制，对涉及经济社会发展全局的重大事项，广泛征询市民和专家的意见；实行“阳光行政”，进一步扩大政务公开范围，提高政府工作透明度，对涉及群众切身利益的有关事项，实行公示、听证制度。严格执行党风廉政建设责任制，加快推进惩治和预防腐败体系建设，加强对行政审批、财政资金分配、专项资金管理、工程招投标等重点领域的监管，坚决纠正和制止土地征收征用、企业重组改制、教育、医疗等方面所存在的损害群众利益的不正之风，依法查处商业贿赂。坚决制止铺张浪费，降低行政成本。加强监察和审计工作，形成用制度规范从政行为的长效机制，从源头上预防和治理腐败，做到实实在在做事，堂堂正正做人，清清白白从政。

做好政府各项工作，离不开各位人大代表、政协委员及社会各界的支持和监督。各级政府要自觉接受人大及其常委会的法律监督和工作监督，自觉接受政协的民主监督，充分听取各民主党派、工商联和无党派人士的意见，及时办理人大代表议案、建议和政协委员提案，充分发挥工会、共青团、妇联等群团组织作用，最广泛地调动全社会一切积极因素，努力开创漳州发展的新局面。

各位代表，新的形势催人奋进，新的征程任重道远。让我们更加紧密地团结在以胡锦涛同志为总书记的党中央周围，高举邓小平理论和“三个代表”重要思想伟大旗帜，在省委、省政府和市委的坚强领导下，团结带领全市人民，奋勇开拓，励精图治，阔步向前，以优异的成绩迎接党的十七大胜利召开，在建设海西港口大市工业强市生态名市和谐漳州的征程上，再创新业绩，再铸新辉煌！
